# Association of biomarkers of inflammation and cell adhesion with lung function in the elderly: a population-based study

**DOI:** 10.1186/1471-2318-13-82

**Published:** 2013-08-07

**Authors:** Antje Kuhlmann, Inga Sif Ólafsdóttir, Lars Lind, Johan Sundström, Christer Janson

**Affiliations:** 1 Department of Medical Sciences, Respiratory Medicine & Allergology, Uppsala University, Uppsala University Hospital, 751 85, Uppsala, Sweden; 2 Department of Medical Sciences, Cardiovascular Epidemiology, Uppsala University, Uppsala University Hospital, 751 85, Uppsala, Sweden

**Keywords:** Lung function, FEV_1_, FVC, COPD, Biomarkers, Gender

## Abstract

**Background:**

Low lung function is associated with increased morbidity and mortality. It is therefore of interest to identify biomarkers that are associated with impaired lung function. The aim of the study was to analyse associations of biomarkers and combinations of biomarkers with lung function in an elderly general population.

**Methods:**

Lung function (FEV_1_ and FVC) and a panel of 15 inflammatory markers from blood samples were analysed in 888 subjects aged 70 years. Biomarkers included cytokines, chemokines, adhesion molecules, C-reactive protein (CRP) and leukocyte count.

**Results:**

Leukocyte count and CRP were independently associated with FEV_1_ after adjustments for other inflammatory markers, sex, BMI, current smoking and pack-years of smoking. In a similar model, leukocyte count and vascular cell adhesion protein 1 (VCAM-1) were the biomarkers that were significantly associated with FVC. Subjects that had both leukocyte count and CRP in the lowest tertile had a FEV_1_ that was 9% of predicted higher than subjects with leukocyte count and CRP in the highest tertile (103±16 vs. 94±21% of predicted, p=0.0002) (mean±SD). A difference of 8% of predicted in FVC was found between subjects with leukocyte count and VCAM-1 in the lowest and highest tertiles, respectively (106±18 vs. 98±19% of predicted, p=0.002).

**Conclusion:**

Leucocyte count, CRP and VCAM-1 were found to relate to poorer lung function. A dose related association was found for the combination leukocyte count and CRP towards FEV_1_ and leukocyte and VCAM-1 towards FVC. This indicates that combination of two biomarkers yielded more information than assessing them one by one when analysing the association between systemic inflammation and lung function.

## Background

Lung function is characterized by slow and irreversible age-related decline. The rate of decline can be accelerated by several factors, e.g. smoking, environmental exposure or lung disease. Lung function is often assessed by measuring forced expiratory volume in 1 second (FEV_1_) and forced vital capacity (FVC). Having a low FEV_1_ has been shown to be related to all-cause mortality [1] and cardiovascular mortality [2], while a low FVC has been associated with higher risk of developing diabetes [3] and myocardial infarction [4]. Low FEV_1_ and FVC are both related to a higher prevalence of hypertension [5] and a higher risk of mortality in patients with chronic heart failure [6]. In one study FVC was found to be more strongly related to survival than FEV_1_[7].

As low lung function is an indicator for increased morbidity and mortality, it is of great interest to identify and validate biomarkers that are associated with impaired lung function. Many serum-biomarkers have been proposed to have relationship with lung function, whereof particularly inflammatory serum-biomarkers such as C-reactive protein (CRP), leukocyte count, fibrinogen, and interleukin (IL)-6 have been related to chronic obstructive pulmonary disease (COPD) and reduced FEV_1_[8-12]. These markers probably reflect both the inflammatory activity in the lungs and an underlying systemic low-grade inflammation. However, the results have varied a lot between studies and it is not possible yet to establish a particular biomarker with adequate relation to lung function. Some studies indicate that assessing combination of biomarkers may be more useful when investigation correlation to lung function [13] or prognosis [14,15].

Several studies indicate a stronger association between biomarker-level and impaired lung function in men than in women [16-19]. It is therefore important to include gender aspects when investigating the association of biomarker-levels to lung function.

The aim of this study was to analyse association between inflammatory biomarkers and lung function in an elderly population and to investigate whether there was a gender-specific difference in this association.

## Methods

### Study population

The design of the Prospective Investigation of Vasculature in Uppsala Seniors study (PIVUS) has been published in detail [20]. In the study, 2,025 subjects aged 70 years were randomly selected from the general population of Uppsala, Sweden. Participation rate was 50.1% (1016 participants). As the participation rate was moderate, cardiovascular disorders and medication in 100 consecutive non-participants were evaluated. The prevalence of cardiovascular drug intake, history of myocardial infarction, coronary revascularisation, antihypertensive medication, statin use, and insulin treatment were similar to in the sample investigated, whereas, the prevalence of diabetes, congestive heart failure and stroke tended to be higher among the non-participants. The study was approved by the Ethic Committee at the University of Uppsala, and all participants gave informed consent.

### Clinical investigations

Participants completed a questionnaire concerning medical history, regular medication use and smoking habits. All subjects were examined the morning after an overnight fast, and no medication or smoking was allowed after midnight. After recording height, weight, abdominal and hip circumference, blood samples were taken and analysed by standard laboratory techniques. Subjects were categorised as never-smokers, ex-smokers or current smokers and pack years of smoking was calculated. BMI was calculated as weight in kilograms divided by the square of the height in metres.

### Spirometry

As previously reported [18,21], spirometry was performed with a Vitalograph Alpha spirometer (Vitalograph Ltd. Buckingham, UK), in accordance with the American Thoracic Society recommendations [22]. The best value of three recordings was used. FEV_1_ and FVC values were expressed as per cent of predicted values, adjusted for age, gender and height. Predicted values for FEV_1_ were based on the European Coal and Steel Union reference values [23].

### Biomarkers

The analysis of cytokines, chemokines and adhesion molecules in blood has been described previously [24]. The panel of analysed biomarkers included CRP, leukocyte count, monocyte chemotactic protein (MCP)-1, interleukin (IL)-1α, IL1β IL-2, IL-4, IL-6, IL-8, IL-10, Interferon gamma (IFN γ), tumour necrosis factor alpha (TNF-α), epidermal growth factor (EGF), vascular endothelial growth factor (VEGF), vascular cell adhesion protein 1 (VCAM-1), intercellular adhesion molecule 1 (ICAM-1), E-selectin, P-selectin, and L-selectin.

High sensitive CRP was measured in human serum by an ultrasensitive particle enhanced immunoturbidimetric assay (Orion Diagnostica, Espoo, Finland) on a Konelab 20 autoanalyser (Thermo Clinical Labsystems, Espoo, Finland). The interassay coefficient of variation was 3.2%.

Cytokines, chemokines and adhesion molecules were analysed on the Evidence® array biochip analyser (Randox Laboratories Ltd., Crumlin, UK) [25]. The intra-assay coefficient of variation was 6.9–15.0% and the interassay coefficient of variation was 8.0–16%. The functional sensitivity for the different inflammatory markers were IL-2, 4.1 ng/L IL-6, 0.3 ng/L, IL-8, 1.5 ng/L; IFN γ, 1.8 ng/L; TNF-α, 1.8 ng/L; MCP-1, 19.4 ng/L; ICAM-1, 18.6 ng/L; VCAM-1, 3.1 ng/L; E-selectin, 3.1 ng/L; P-selectin, 11.2 ng/L; L-selectin, 32.8 ng/L; CRP, 0.1 mg/L; and, leukocyte count, 0.2 x 10^9^.

The informative value of the levels of IL-1α, IL-1β, IL-4 and IL-10 was limited, because the sensitivity of the applied Evidence® array appeared insufficient for these biomarkers, with more than half of the values below detection limit. These variables were therefor not included in the analyses.

### Cardiovascular comorbidity

The following comorbidities were included in the analyses: hypertension (using medication against hypertension, myocardial infarction and stroke (having been hospitalised because of myocardial infarction or stroke).

### Statistics

Statistical analyses used STATA 12 software (Stata Corp., College Station, Texas, USA). All variables, except FEV_1_, FVC and leukocyte count, were log-transformed to obtain a normal distribution. Simple linear regression was used to assess the association of biomarkers with FEV and FVC. The independent relationship between biomarkers that were significantly associated with FEV_1_ or FVC in the univariate analyses and lung function were analysed by multiple linear regression with adjustment for gender, BMI, current smoking status, and pack-years of smoking. As there were 84 missing values for pack-years, STATA mi-commands were used for multiple imputation of the missing data. Models were also made with adjustments for cardiovascular comorbidity. The biomarkers were standardised to one SD in all regression models. Biomarkers that were significantly associated with lung function in the final multivariable model were grouped into tertiles in order to study the added value of analysing two biomarkers simultaneously, and in order to accommodate non-linear associations. Analyses stratified for gender were performed, and analyses of interactions between gender and biomarker levels were done. *P*<0.05 was considered statistically significant.

## Results

### Characteristics of the study population

Data on spirometry was available in 888 participants, and the characteristics of the study population are presented in Table 1.

**Table 1:** Clinical characteristics of participants (mean ± SD and %)

	**Men (n=432)**	**Women (n=456)**	**All subjects (n=888)**
BMI (kg/m^2^)	27.1±3.6	27.0±4.8	27.0 ± 4.3
Current smokers	10	11	10
Ex-smokers	48	35	41
Never-smokers	42	54	48
Hypertension	31	32	32
Myocardial infarction	10	3	7
Stroke	4	2	3
FEV_1_ (L)	2.88±0.62	2.02±0.41	2.44±0.68
FEV_1_ % predicted	96±20	101±19	99±20
FVC (L)	3.85±0.70	2.60±0.49	3.21±0.87
FVC % predicted	95±16	108±18	102±18
Leukocyte count (x10^9^/L)	5.8±1.5	5.5±1.4	5.7±1.5
CRP (mg/L)	1.2 (1.1-1.4)	1.4 (1.3-1.5)	1.3 (1.2-1.4)
MCP-1 (ng/L)	368 (355–381)	366 (353–380)	367 (358–377)
IL-2 (ng/L)	4.6 (4.2-4.9)	5.0 (4.6-5.4)	4.8 (4.5-5.0)
IL-6 (ng/L)	7.0 (6.0-8.1)	5.8 (5.0-6.8)	6.4 (5.7-7.1)
IL-8 (ng/L)	6.3 (5.9-6.7)	6.6 (6.2-7.0)	6.4 (6.2-6.7)
INF γ (ng/L)	1.8 (1.7-1.9)	1.9 (1.8-2.0)	1.8 (1.8-1.9)
TNFα (ng/L)	4.2 (4.0-4.4)	4.0 (3.8-4.2)	4.1 (3.9-4.2)
EGF (ng/L)	20.6 (18.7-22.8)	24.2 (22.0-26.5)	22.4 (20.9-23.9)
VEGF (ng/L)	161 (150–174)	191 (177–206)	176 (167–185)
VCAM-1 (μg/L)	540 (529–552)	508 (497–520)	524 (515–532)
ICAM-1 (μg/L)	346 (339–354)	354 (345–362)	350 (345–356)
E-selectin (μg/L)	15.0 (14.5-15.5)	13.9 (13.3-14.4)	14.4 (14.0-14.8)
P-selectin (μg/L)	99.2 (96.0-102)	95.7 (93.2-98.3)	97.4 (95.4-99.5)
L-selectin (μg/L)	688 (676–700)	731 (718–744)	709 (701–718)

### Biomarker and lung function

#### Univariate analysis

The association between values for 15 biomarkers and FEV_1_ and FVC was analysed with linear regression. Leukocyte count, CRP and E-selectin had a negative and EGF a positive association with the two lung function parameters (Table 2). ICAM-1 and VCAM-1 were negatively associated with FEV_1_ and FVC, respectively. Otherwise, no major associations were found between the different biomarkers and lung function.

**Table 2:** **Association between inflammatory markers and FEV**_**1 **_**or FVC when analysed by simple linear regression analysis**

	**FEV**_**1 **_**(% predicted)**	**p-value**	**FVC (% predicted)**	**p-value**
Leukocyte count	−3.39 (−4.68, -2.11)	<0.0001	−2.44 (−3.63, -1.26)	<0.0001
CRP	−2.67 (−3.96, -1.38)	<0.0001	−1.51 (−2.70, -0.32)	0.01
EGF	1.42 (0.12, 2.71)	0.03	1.65 (0.47, 2.84)	0.006
VCAM-1	−1.17 (−2.46, 0.13)	0.08	−2.14 (−3.33, -0.96)	<0.0001
ICAM-1	−2.49 (−3.78, -1.20)	<0.0001	−1.16 (−2.35, 0.03)	0.06
E-selectin	−1.63 (−2.92, -0.33)	0.01	−1.84 (−3.03, -0.65)	0.002
MCP-1	0.44 (−0.86, 1.74)	0.50	0.51 (−0.69, 1.70)	0.41
IL-2	0.20 (−1.10, 1.50)	0.76	0.17 (−1.02, 1.37)	0.78
IL-6	−0.19 (−1.51, 1.12)	0.77	−1.01 (−2.22, 0.19)	0.10
IL-8	−0.47 (−1.78, 0.83)	0.48	−0.80 (−2.00, 0.39)	0.19
INF γ	0.13 (−1.18, 1.43)	0.85	−0.20 (−1.40, 0.99)	0.74
TNFα	0.01 (−1.29, 1.31)	0.99	−0.74 (−1.94, 0.45)	0.22
VEGF	−0.34 (−1.64, 0.96)	0.61	0.55 (−0.65, 1.74)	0.37
P-selectin	−0.75 (−2.05, 0.55)	0.26	−0.54 (−1.73, 0.66)	0.34
L-selectin	0.78 (−0.52, 2.08)	0.24	0.53 (−0.66, 1.73)	0.38

Values are β-coefficient (95% CI), in % per 1 SD change in biomarker. Markers that are significantly associated with lung function are presented in the first part of the table.

#### Multivariate analyses

The independent associations between different biomarkers and lung function were studied with multiple linear regression. No significant association between E-selectin and FEV_1_ or FVC was found after adjusting for the other biomarkers that were significant associated with lung function in the univariate model. Leukocyte count and CRP remained statistically associated with FEV_1_ after further adjustments for sex, BMI, current smoking and pack years (Table 3). In a similar model leukocyte count and VCAM-1 were the biomarkers significantly associated with FVC. FEV_1_ and FVC were also significantly associated with female gender and pack years, while current smoking and BMI were independently associated with FEV_1_ and FVC, respectively (Table 3). The associations above remained significant after further adjustment for hypertension, myocardial infarction and stroke.

**Table 3:** Association between inflammatory markers and lung function

	**FEV**_**1 **_**% predicted**	**p-value**	**FVC % predicted**	**p-value**
Leukocyte count	−1.64 (−3.03, -0.24)	0.02	−1.77 (−2.96, -0.58)	0.005
CRP	−1.71 (−3.09, -0.32)	0.02	-	
EGF	1.23 (−0.002, 2.46)	0.05	1.05 (−0.06, 2.15)	0.06
VCAM-1*	-		−1.28 (−2.45, -0.11)	0.03
ICAM-1**	−0.97 (−2.30, 0.36)	0.13	-	
Women	3.42 (0.90-5.94)	0.008	11.8 (9.54, 14.1)	<0.0001
BMI	0.05 (−0.26,0.36)	0.70	−0.29 (−0.56, -0.02)	0.04
Current smoking	−6.61 (−11.2,-1.99)	0.005	−0.21 (−4.27, 3.86)	0.96
Pack years	−0.30, (−0.41, -0.18)	<0.0001	−0.15 (−0.24, -0.04)	0.005

All estimates are adjusted for each of the included inflammatory markers, pack years, current smoking status, gender, and BMI.

Values are β-coefficient (95% CI), in % per 1 SD change in biomarker.

* Only included in model with FVC as dependent variable.

** Only included in model with FEV_1_ as dependent variable.

**Figure 1:**
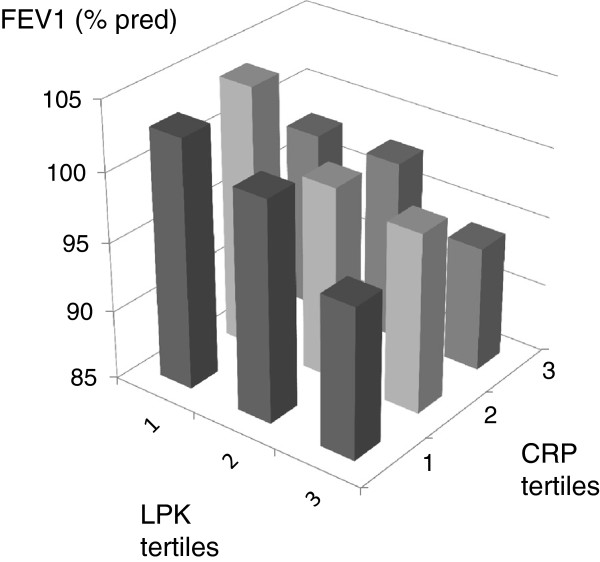
**FEV**_**1 **_**(% predicted) in participants divided by tertile of leukocyte count (<4.1, 4.1-6.0, >6.0 x10**^**9**^**/L) and tertitles of C-reactive protein (CRP) (<0.78, 0.78-1.8, >1.8 mg/L).**

**Figure 2:**
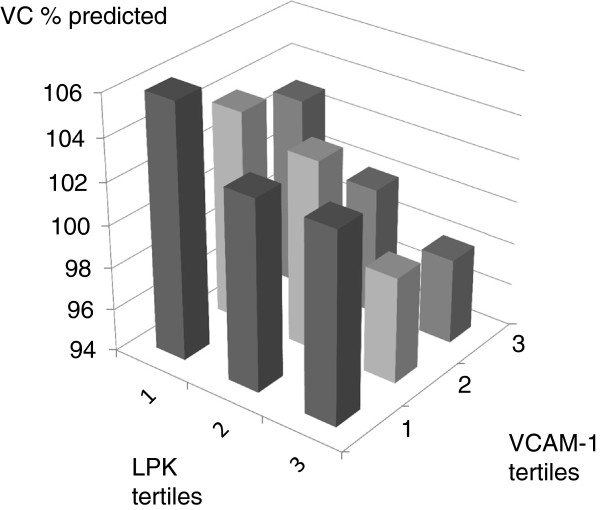
**FVC (% predicted) in participants divided by tertile of leukocyte count (<4.1, 4.1-6.0, >6.0 x10**^**9**^**/L) and tertitles of VCAM-1 (<470, 470–570, >570 μg/L).**

Figures 1 and 2 present the mean FEV_1_ and FVC when combining the two biomarkers that were predominantly associated with each lung function variable. Subjects that had leukocyte count and CRP in the lowest quartile had a FEV_1_ that was 9% of predicted higher than subjects with leukocyte count and CRP in highest tertile (103±16 vs. 94±21% of predicted, p=0.0002) (mean±SD). A difference of 8% of predicted in FVC was found between subjects leukocyte count and VCAM-1 in the lowest and highest tertiles respectively (106±18 vs. 98±19% of predicted, p=0.002) (mean+SD).

### Gender differences

**Figure 3:**
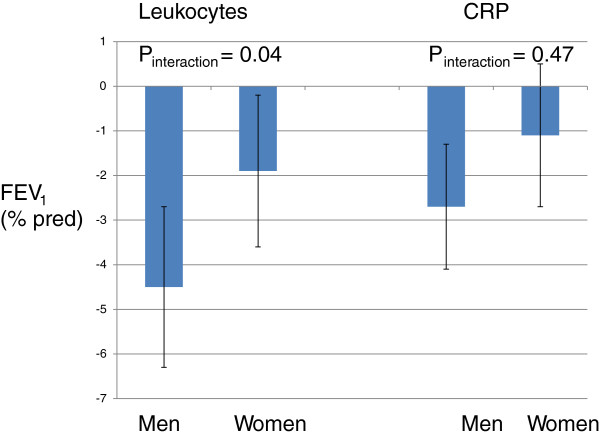
**Estimates (95% CI) of associations between biomarkers and FEV**_**1 **_**in men and women.**

**Figure 4:**
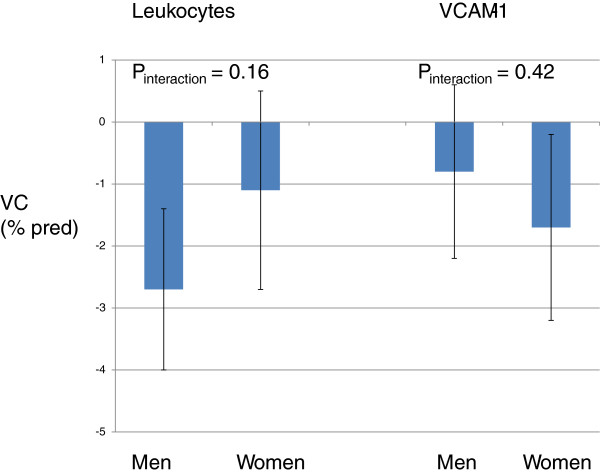
Estimates (95% CI) of associations between biomarkers and FVC in men and women.

The association between leukocytes and FEV_1_ was statistically stronger in men than in women (Figure 3). The association between CRP and FEV_1_ and leukocytes and FVC was numerically stronger in men than women while the opposite was found for the association between VCAM-1 and FVC, but none of these differences were statically significant when analysed with test of interaction (Figures 3 and 4).

## Discussion

In this study, a large number of biomarkers and their association to lung function in a population-based sample of 70-year old men and women were analysed. A high number of circulating leukocytes and a high level of CRP were independently related to lower FEV_1_, while a high leucocyte count and high level of circulating VCAM-1 was associated with a lower FVC.

The relationship between higher levels of inflammatory biomarkers and lower lung function in the present study are in accordance with what has been found in many previous investigations [11,16-19,26-29]. However, the results of these studies have been very varied regarding which particular biomarker was related to which specific lung function parameter. We found a significant association between CRP, leukocyte count and lung function. This in accordance with several longitudinal studies that have found an association between the rate of FEV_1_ decline and leukocyte count [29] and CRP [19,30]. On the other hand we did not find an association between lung function and IL-6 which is in contrast what has been found in some other studies [11,16]. The lack of association between Il-6 and lung function in the present study is difficult to explain, as IL-6 and CRP are closely related in the inflammatory pathway, IL-6 stimulating the hepatic production of CRP.

In the present study circulating levels of ICAM-1 and particularly VCAM-1 were negatively related to lung function. To some extent this finding is not surprising as both ICAM-1 and VCAM-1 facilitate the recruitment and migration of inflammatory cells such as neutrophils from the blood to the airway walls. Previous studies have found higher levels of circulating adhesion molecules in COPD patients [31] and having high levels of ICAM-1 and VCAM-1 was related to higher airway resistance in asthma in one study [32]. We have, however, been unable to find previous general population studies showing an association between high levels of circulating adhesion molecules and lung function.

The combination of leukocyte count and CRP showed a dose response trend towards lower FEV_1_ whereas the combination of leukocyte count and VCAM-1 showed a similar trend towards FVC. This supports the idea that assessing a combination of biomarkers may be more informative than assessing them one by one. In the Woman’s Health and Aging Studies, a population based study on about thousand elderly women; the combined highest levels of IL-6 and CRP were associated with the lowest levels of FEV_1_ and FVC [13]. A combination of several serum biomarkers has also been used to improve the prediction of mortality and risk for comorbidities in COPD. In the ECLIPSE study, the addition of a panel of 10 inflammatory biomarkers to the clinical variables improved the ability to predict mortality in COPD significantly [14]. The data of two large Danish population studies indicate that simultaneously elevated levels of CRP, fibrinogen and leukocyte count are associated with a two- to fourfold risk of major comorbidities (e.g. myocardial infarction, lung cancer) in COPD [15].

In the present study smoking and BMI were as expected associated with lung function. Both these variables are also associated with systemic inflammation [33,34]. It was therefore important to adjust for smoking status and BMI in our final models. Inflammatory biomarkers like CRP are also associated with cardiovascular diseases such as intracerebral hemorrhage [35] and myocardial infarctions [36]. In the present the associations between leukocyte count and CRP and leukocyte count and VCAM-1 remained statically significant also after adjustment for cardiovascular comorbidity.

The association between leukocyte count and FEV_1_ was stronger in men than in women. This result is in accordance with previous publications from our groups showing that the association between lung function and systemic inflammation is stronger in men than in women [16-19]. The biological explanation for this gender difference is largely unknown. There are, however, differences between the clinical manifestation and pathophysiology of lung disease in men and women [37,38]. Women with COPD have more dyspnea, anatomically smaller airway lumens with disproportionately thicker airway walls and less extensive emphysema.

The strength of this study was that data were collected from a general population and analysed with high-quality, standardised methods. A wide range of biomarkers and their relation to lung function was analysed simultaneously. There are, however, also limitations of the study that deserve mention. First, the study had a moderate participation rate, even though an analysis of non-participants revealed that the present sample was representative for the total population. Second, post-bronchodilator measurements of lung function were not performed and it was therefore not possible to assess the association between systemic inflammation and COPD in a reliable manner. Third, the sensitivity of the method for measuring IL-1α, IL-1β, IL-4 and IL-10 was insufficient, as many values were under detection limit. It should also be noted that the associations found were fairly weak which indicates that the analysed biomarkers are not clinically useful at present.

Low lung function in elderly is associated with a shorter life expectancy [1,2] and morbidity [3-5]. In a another analysis of the present population we also found that low lung function was related to autonomic dysfunction [21], which could contribute to the association between low lung function and cardiovascular comorbidity. Systemic inflammation is also a likely contributor to the association between low lung function and morbidity [14,15]. As the present study was cross-sectional the cause and effect relationship between systemic inflammation, lung function and morbidity has to be explored in more detail in longitudinal studies.

## Conclusion

Leucocyte count, CRP and VCAM-1 were found to relate to poorer lung function. A dose related association was found for the combination leukocyte count and CRP towards FEV_1_ and leukocyte and VCAM-1 towards FVC. This indicates that combination of two biomarkers yielded more information than assessing them one by one when analysing the association between systemic inflammation and lung function. The association between systemic inflammation and lung function maybe stronger in men than women.

## Abbreviations

FEV1: Forced expiratory volume in one second; FVC: Forced vital capacity; CRP: C-reactive protein; BMI: Body mass index; VCAM-1: Vascular cell adhesion protein 1; COPD: Chronic obstructive pulmonary disease; PIVUS: Prospective Investigation of Vasculature in Uppsala Seniors; MCP: Monocyte chemotactic protein; IL: Interleukin; IFN γ: Interferon gamma; TNF-α: Tumour necrosis factor alpha; EGF: Epidermal growth factor; VEGF: Vascular endothelial growth factor; ICAM-1: Intercellular adhesion molecule 1.

## Competing interests

The authors declared that they have no competing interests.

## Authors’ contributions

AK, ISO and CJ analysed the data and wrote the manuscript. LL and JS conceived and supervised the study. All authors critically revised the manuscript. All authors read and approved the final manuscript.

## Pre-publication history

The pre-publication history for this paper can be accessed here:

http://www.biomedcentral.com/1471-2318/13/82/prepub
